# *Escherichia coli* Strains Originating from Raw Sheep Milk, with Special Reference to Their Genomic Characterization, Such as Virulence Factors (VFs) and Antimicrobial Resistance (AMR) Genes, Using Whole-Genome Sequencing (WGS)

**DOI:** 10.3390/vetsci12080744

**Published:** 2025-08-08

**Authors:** Theodora Skarlatoudi, Glykeria-Myrto Anagnostou, Vasileios Theodorakis, Loulouda Bosnea, Marios Mataragas

**Affiliations:** 1 Department of Dairy Research, Institute of Technology of Agricultural Products, Hellenic Agricultural Organization “DIMITRA”, Katsikas, 45221 Ioannina, Greece; theodoraskarlatoudi@hotmail.com (T.S.); anmirto@yahoo.gr (G.-M.A.); bosnea@elgo.gr (L.B.); 2 General Agricultural Cooperative of Ioannina “Farmers’ Union”, Krya, 45500 Ioannina, Greece; btheodorakis@gasi.gr

**Keywords:** ewes, whole-genome sequencing, bioinformatics, genetic features, microorganisms, food-borne pathogens, *Escherichia coli*, molecular microbiology

## Abstract

The objective of this work was to deliver a comprehensive genetic characterization of a collection of *E. coli* strains isolated from raw sheep milk. To complete our purpose, the technique of whole-genome sequencing, coupled with bioinformatics and phenotypic characterization of antimicrobial resistance, was performed. These Gram-negative, facultative anaerobic bacteria belong to the family *Enterobacteriaceae*, together with other intestinal pathogens, such as *Shigella* spp. and *Salmonella* spp. Genetic analysis was carried out on all strains (phylogram, sequence types, VFs, AMR genes, and pangenome). The results showed the presence of various genetic traits that are related to virulence factors contributing to their pathogenic potential. In addition, genes conferring resistance to antibiotics were also detected and confirmed using phenotypic tests. Finally, the genome of the *E. coli* strains was characterized by the presence of several mobile genetic elements, thus facilitating the exchange of various genetic elements, associated with virulence and antimicrobial resistance, within and beyond the species, through horizontal gene transfer. Contaminated raw sheep milk with pathogenic *E. coli* strains is particularly alarming for cheese production in artisan dairies.

## Simple Summary

Mastitis in farm animals constitutes a significant problem for livestock farms with an impact on their sustainability, affecting production costs and milk quality. The main causative agents of the disease are pathogenic bacteria (e.g., staphylococci, streptococci, *Escherichia coli*, etc.), originating from the environment, milking parlor, equipment, personnel, etc. These colonize the udder and cause infection after invasion. The result of this infection is reduced milk production and milk of low quality. The aim of this work was to molecularly characterize the *Escherichia coli* strains isolated from raw sheep milk and determine their virulence and antimicrobial resistance potential. The isolated strains were characterized at the molecular level, gaining insights into their genetic capacity and background. The results of this study will enhance the currently available knowledge, as there is not much literature available in this area.

## 1. Introduction

*E. coli* strains are Gram-negative, facultative anaerobic microorganisms which colonize the lower gastrointestinal tract of humans and animals and belong to the *Enterobacteriaceae* family. In dairy farms, *E. coli* is found in organic matter such as bedding and manure, subsequently infecting the mammary gland through contact with the environment [1,2].

*E. coli* is distinguished in two groups, namely the intestinal pathogenic *E. coli* (IPEC) and the extraintestinal pathogenic *E. coli* (ExPEC), and infection may result in serious illness. The most known pathotypes of the IPEC group are the enteroaggregative *E. coli* (EAEC), enteropathogenic *E. coli* (EPEC), enteroinvasive *E. coli* (EIEC), enterohemorrhagic *E. coli* (EHEC), and enterotoxigenic *E. coli* (ETEC). The *E. coli* genome is well known for its plasticity, allowing the exchange of VF- and/or AMR-related genes through the different mobile genetic elements (MGEs) such as plasmids, insertion sequences, etc., a phenomenon called horizontal transfer, leading to pathotypes known as hybrids, which constitute an emerging type of *E. coli* [3,4,5,6,7,8,9,10]. These hybrid strains carry VFs otherwise found in other *E. coli* pathotypes like EAEC, EPEC, EIEC, EHEC, ETEC, or ExPEC [11,12].

Most genetic information on *E. coli* strains found in dairy farms originates from cattle [3,4,5,6,7,8,13,14,15] but little is known about *E. coli* found in raw ewes’ milk. The knowledge of the pathogenic traits (VFs and AMR) of *E. coli* isolated from raw sheep milk is limited. Since raw sheep milk is mainly directed to the production of cheese, dairy ewes may represent a potential source of *E. coli*; however, at present, this risk is not clear. Therefore, the following research question was made: “Could raw milk of dairy sheep be a potential *E. coli* reservoir, and what are the pathogenic traits of these strains?”. Consequently, the research objectives of the present study were to (a) study the *E. coli* strains isolated from raw sheep milk with the goal of enriching the presently insufficient data on the genetic features of this microorganism derived from the ewes’ milk, which is widely used in the production of artisanal, Protected Designation of Origin (PDO), and raw cheeses, and (b) determine the AMR and virulence potential of the isolated *E. coli* strains, using bioinformatics, phenotypic (AMR), and genotypic (VFs and AMR) methods.

## 2. Materials and Methods

### 2.1. Microbial Dataset

A farm located in the Epirus region was sampled over a year (12 months), taking four bulk tank milk samples (50 mL each) at each visit (two visits per month, i.e., every 15 days). The samples were cultured on the selective agar CHROMagar^TM^ Mastitis (Bioprepare Microbiology, Athens, Greece, 020366) by streaking 0.01 mL with a bacteriological loop according to National Mastitis Council instructions, and incubated at 37 °C for 24 h. Based on the color of the colonies, all suspected colonies were isolated, purified on non-selective media and kept frozen at −80 °C in Brain Heart Infusion (BHI) broth (Condalab, Madrid, Spain, 1400) supplemented with 30% glycerol (Penta Chemicals, Prague, Czech Republic, 14530–11000PE) as cryoprotectant agent until their identification. The strains that are discussed in this paper refer only to *E. coli*. The microbial dataset consisted of 15 strains isolated on different sampling dates (Table S1). Before use, the strains were revived twice in the respective medium (BHI broth) for 24–48 h at 37 °C.

### 2.2. Whole-Genome Sequencing and Assembly

Genomic DNA was extracted from the presumably identified *E. coli* strains, which were then sequenced using the Illumina NovaSeq 6000 (San Diego, CA, USA) short-read (paired-end, 2 × 150 bp) platform [16], generating adapter-free raw fastq files (on-board). The in-silico analysis of the adapter-free raw fastq reads, including quality control of the raw reads, polishing and de novo assembling of the raw reads into contigs, organization of the contigs into scaffolds, orientation of the scaffolds, quality assessment of the scaffolds (contamination and completeness), and evaluation of misassemblies after scaffolding, was performed as described in the work of Apostolakos et al. (2023) [17]. The reference genome *Escherichia coli* DSM30083 (indicated as the closest reference strain to our strains by Bionumerics and GTDB-Tk), downloaded from the NCBI database (https://www.ncbi.nlm.nih.gov/, accessed on 10 April 2024), was used for scaffolding and orientation of the scaffolds. High-quality assemblies of the draft *E. coli* genomes (completeness ≥ 95%, contamination ≤ 5%, heterogeneity 0%, number of contigs/scaffolds below 200, the shortest contig length that needs to be included for covering 50% of the genome, i.e., N50 > 30,000 bp, and genome size 5 Mbp ± 0.5 Mbp), evaluated with the CheckM v1.0.18 [18] and QUAST v5.2.0 [19] tools, were further analyzed with advanced computational and bioinformatic tools.

### 2.3. Bioinformatic Analysis

A preliminary molecular characterization (taxonomy, virulence genes and virulence islands, antimicrobial resistance genes, serotype, plasmids, and phages) of the isolated species was carried out using the Bionumerics v8.1 software and the *E. coli* functional genotyping module (bioMérieux, Sint-Martens-Latem, Belgium). In addition, the following bioinformatic tools were employed: the Genome Taxonomy Database Toolkit (GTDB-Tk) [20], Type (Strain) Genome Server (TYGS) (https://tygs.dsmz.de/, accessed on 12 April 2024) [21], and Average Nucleotide Identity (ANI) values, calculated with the OrthANI tool (https://www.ezbiocloud.net/, accessed on 12 April 2024) [22], for species confirmation; the PROKKA v1.14.5 [23] and eggnog-mapper v2.1.12 [24] for genome and functional annotation, respectively; the PathogenFinder v1.1 [25] for the identification of strains’ pathogenic capacity in humans; the abricate v1.0.1 [26], AMRFinderPlus v3.11.26 [27] STARAMR 0.10.0 [28], and abriTAMR v1.0.15 [29] along with the reference databases VFDB v2.0 [30], *Escherichia coli* O-groups and H-types (EcOH) [31], ecoli_vf [32], MobileElementFinder v1.1.2 [33], ResFinder v4.7.2 [34], Antibiotic Resistance Gene Annotation (ARGANNOT) [35], Comprehensive Antibiotic Resistance Database (CARD) [36], Microbial Ecology Group Antimicrobial Resistances (MEGARes) [37], National Center for Biotechnology Information (NCBI) resistance gene [38], and PlasmidFinder v2.2 [39], for the identification of VFs, MGEs, antimicrobial resistance genes (ARGs), plasmid replicons, and in silico serotyping of *E. coli*.

Multi-Locus Sequence Typing (MLST) v2.0 [40], FimTyper v1.0 [41], CHTyper v1.0 [42], SerotypeFinder v2.0 [43], and cgMLSTFinder v1.2 [44,45] were used for sequence type (ST) classification with the Achtman MLST scheme [46], FimH and FimC typing, serotype identification, and core-genome multi-locus sequence typing, respectively. Pangenome analysis and core genome alignment were carried out with the Roary v3.11.2 [47] software. Proteins were grouped into the same family if their amino acid sequence similarity was ≥95%. If a gene was present in at least 99% of strains, then it was included in the core genome. The kSNP v3.0 algorithm [48] and FastTree v2.1 tool [49] were used for the determination of phylogenetic relationships, and the Interactive Tree of Life (iTOL) v6 [50] for the visualization of the resulting tree. Finally, the ClermonTyping web server (http://clermontyping.iame-research.center/, accessed on 12 April 2024) [51,52] was employed for the identification of phylogroups (phylotyping). The Integrated Prokaryotes Genome and pangenome Analysis (IPGA) service v1.09 (https://nmdc.cn/ipga/, accessed on 12 April 2024) [53], the Center for Genomic Epidemiology (CGE) services (http://www.genomicepidemiology.org/services/, accessed on 12 April 2024), and the European public Galaxy server (https://usegalaxy.eu/, accessed on 12 April 2024) [54], were also used for the analyses. The concatenation and summarization of the results obtained from the different tools were achieved with the hAMRonization tool [55]. The genome map was built using the Brick webtool (https://brick.ink/, accessed on 5 May 2025) [56], which is based on BLAST DBv5 Ring Image Generator (BRIG) [57], Basic Local Alignment Search Tool (BLAST) [58], geNomad for the identification of mobile genetic elements [59], and abritAMR for the identification of antimicrobial resistance genes [29]. Heatmaps and summary graphs were generated from the Roary’s output file “genes’ presence/absence” using R software for Windows v4.4.3 [60,61]. The BPGA v1.3 [62] tool was used to visualize the distribution/frequency of the annotated Clusters of Orthologous Groups/Genes (COGs) and Kyoto Encyclopedia of Genes and Genomes (KEGG) categories. COG and KEGG heatmaps were built using the ImageGP webtool (https://www.bic.ac.cn/BIC/, accessed on 5 May 2025) [63]. All tools were executed using the default settings of the parameters.

### 2.4. Antimicrobial Susceptibility Testing

For validation of the genomic analysis related to the antimicrobial resistance genes found in the *E. coli* genomes, selected strains were tested for antimicrobial resistance using the Sensititre™ MIC (Thermo Fisher Scientific, Waltham, MA, USA) method using the broth microdilution MIC strategy, according to the manufacturer’s instructions. The antimicrobial susceptibility testing (AST) plates used were the Sensititre™ Mastitis CMV1AMAF Vet AST Plate (Thermo Fisher Scientific). The strains were categorized as susceptible (S), resistant (R), or intermediate (I) based on the European Committee on Antimicrobial Susceptibility Testing (EUCAST) Breakpoint Table v14.0 for *E. coli* (https://www.eucast.org/clinical_breakpoints/, accessed on 28 February 2024), Clinical and Laboratory Standards Institute (CLSI) Ed34 Breakpoints (https://clsi.org/resources/breakpoint-implementation-toolkit/, accessed on 28 February 2024).

## 3. Results and Discussion

### 3.1. Genome Assembly and Annotation

In the present work, fifteen *E. coli* strains isolated from raw sheep mastitis milk were subjected to whole-genome sequencing. The quality metrics of the genome assembly in terms of genome completeness, contamination, heterogeneity, number of contigs/scaffolds, N50, and genome size are presented in Table 1. Genome characteristics and annotations, after removing the low-quality draft genomes, are shown in Table S2. Two genomes (S16 and S25) had completeness below 95% (87.40 and 88.38, respectively, coupled with a high percentage of genome heterogeneity of 30%) and one genome (S19) had contamination above 5% (8.51%). In addition, the number of contigs/scaffolds was high, resulting in either low N50 (S16) or genome size outside the respective limits (S25 < 4.5 Mbp or S19 > 5.5 Mbp). Therefore, these strains were excluded from any bioinformatic analysis. One genome (S40) presented a high number of contigs/scaffolds (764), leading to low N50, which was below the threshold of 30,000 bp. Thus, the S40 strain was also omitted from the downstream analysis.

In the remaining eleven high-quality draft genomes, the genome size of the *E. coli* strains varied from 4.66 to 5.29 Mbp with a GC content from 49.33 to 50.77%. The number of contigs/scaffolds ranged between 2 and 156, with N50 between 37,409 and 4,517,476 bp. The number of coding DNA sequences (CDS) found in each *E. coli* strain and the number of genes called were in the range of 4212–4680 and 4288–4758, respectively. Lastly, the repeat regions were one to four, while the RNA genes in each strain were as follows: one to nine ribosomal RNAs (rRNA), 73 to 86 transfer RNAs (tRNA), and one transfer-messenger RNA (tmRNA) (Tables S1 and S2).

### 3.2. Phylogenetic Analysis and Genotyping

**Figure 1:**
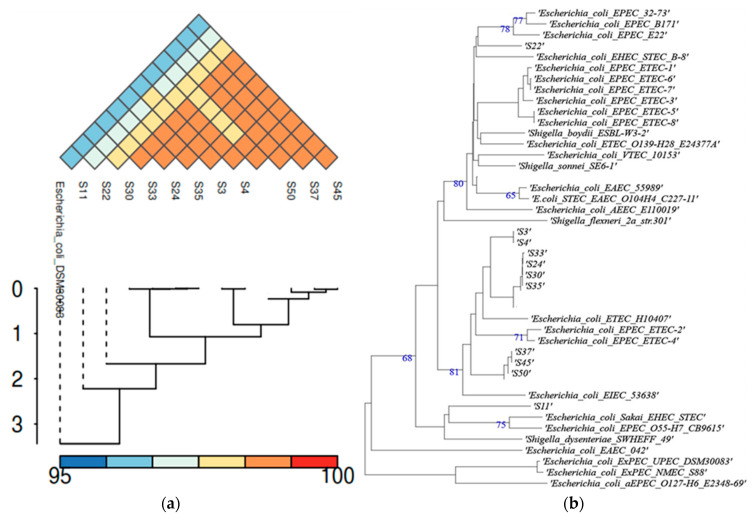
Comparative genomic analysis of the *E. coli* strains: (**a**) heatmap and dendrogram of their ANI values, including the reference *E. coli* DSM30083 strain; and (**b**) WGS-based phylogram as determined by TYGS, including various reference strains. The TYGS platform, during the analysis, automatically selects the reference strains for constructing the phylogram.

The bionumerics *E. coli* module plugin, GTDB-Tk, and TYGS classified all strains as *E. coli* species. ANI values confirmed that all strains belong to the same species since all values between the reference *E. coli* DSM30083 and the query strains, as well as among the query strains themselves, were above 96% (96.56 to 100.00%), i.e., above the 95–96% threshold that is typically used for species-level delineation (Figure 1a). This species-level identification was documented through the phylogenetic analysis (Figure 1b) and the digital DNA–DNA hybridization (dDDH) values, both estimated during the genome-based taxonomy by TYGS. dDDH values ranged from 73.6 to 100.0% between the reference and the query strains, as well as among the query strains. This range of values was above the threshold of 70%, which is recommended to delineate species.

Genotyping results (phylotype, serotype, MLST, cgMLST, and CH genes) are shown in Table 1, indicating the genetic diversity of the *E. coli* strains isolated from raw sheep mastitis milk. All strains were recognized as human pathogenic strains with a high probability of being pathogens (84.2 to 94.2%). The analyzed draft genomes of the *E. coli* strains were categorized into five distinct serotypes (O179:H40, O169:H46, O18ac:H7, O107:H27, and H26), core-genome multilocus sequence types (23273, 34239, 148610, 56618, and 23653), and fumC/fimH type (11/137, 23/31, 95/31, 11/27, and 11/54), while the identified sequence types (STs) were four (ST10, ST1131, ST351, and ST4977). Most of the strains (81.82%) belonged to the phylogroup A. One strain (S22) was classified into phylogroup B1, and one strain (S11) into phylogroup E. The dominance of phylogroup A confirmed the *E. coli* characterization as environmental pathogen since the phylogroups A and B1 are very common in mastitis of dairy animals [10,64,65]. Similarly, the ST10 is the most frequently isolated MLST [3,4,5,8,10,66].

### 3.3. Pangenome Analysis

**Figure 2:**
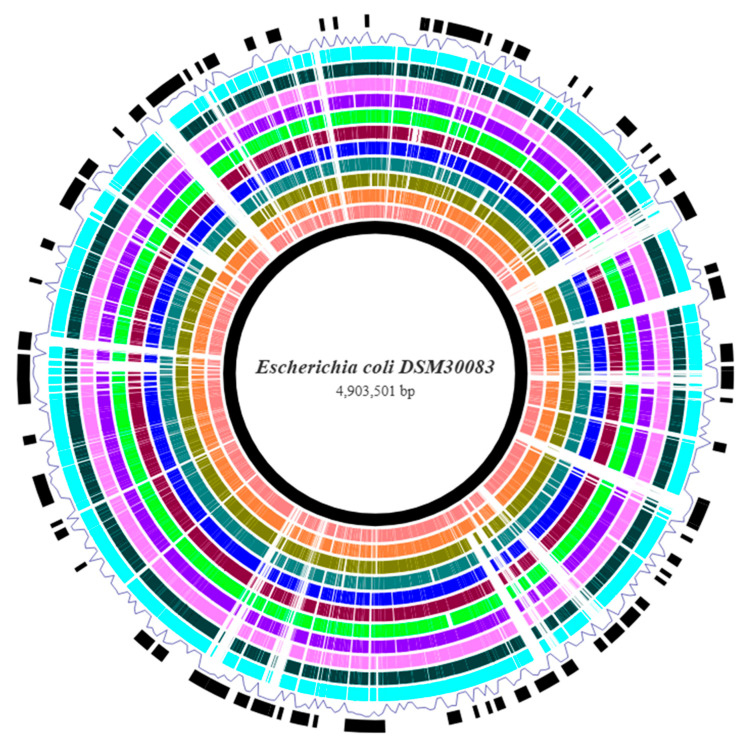
Genome map of the *E. coli* strains compared to the reference genome *E. coli* DSM30083 after their alignment. From the inner toward the outer circle: reference (black color), S3, S4, S11, S22, S24, S30, S33, S35, S37, S45, and S50 (cyan color). The ring drawn as a line (blue color) is the geNomad ring, which shows the prediction scores for horizontal gene transfer or integrated phage regions. When the score exceeds a threshold value, the respective region of the genome is annotated (solid black squares or rectangles). White regions (gaps) in the query genomes mean that this region is absent from the examined genome.

Visualization of the *E. coli* prokaryotic genomes against the reference genome was attained using the Brick webtool, which constructs a BRIG-like interactive data visualization for bacterial genome comparisons. The query genomes were aligned toward the reference for constructing the genome map, which highlights the genetic differences that exist between the different strains (Figure 2). A noteworthy observation from this figure is the relatively frequent annotation of regions with a geNomad score exceeding the threshold value for horizontal gene transfer regions (plasmids and other mobile genetic elements), supporting the well-established opinion that *E. coli* genome is characterized by great plasticity, which helps its genetic evolution, showing significant diversity from non-pathogenic (commensal, avirulent) to extremely pathogenic strains. As this genome visualization and comparison functioned as a fast-screening process to identify any conserved and divergent regions, pangenome analysis was performed for a deeper insight regarding the core and accessory genomes.

**Figure 3:**
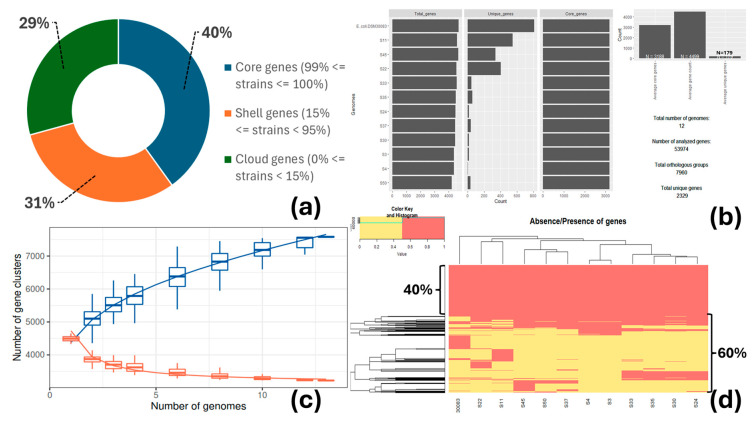
Pangenome analysis of the *E. coli* strains, including the reference *E. coli* DSM30083 strain: (**a**) proportion of the core, shell, and cloud genes. The graph does not depict the number of soft-core genes (95% ≤ strains < 99%) because it was equal to zero; (**b**) distribution of the core and accessory-unique genomes across the studied *E. coli* strains; (**c**) alteration of the size of pan (blue boxes) and core (red boxes) genes as a function of the number of added sequenced genomes; and (**d**) heatmap of the presence (red) and absence (yellow) of the 7960 genes (rows) across the 12 genomes (columns) and separation of core (40%, present in all strains) and accessory-unique (60%, present in some strains and absent in others) genes.

The core genome encompassed 3188 genes present in at least 99% of the strains (*n* = 12, including the reference strain) and represented 40% of the total number of genes (7960) (Figure 3a,b). The size of the accessory genome was remarkable. It is separated into shell genes (15% ≤ genes present in strains < 95%; 2443 or 31%) and cloud or unique genes (0% ≤ genes present in strains < 15%; 2329 or 29%), which sum up to 60% in total. The number of gene clusters present in the pangenome (core and accessory genomes) increased with the number of genomes included in the analysis, whereas the number of gene clusters inside the core genome decreased, reaching a plateau (Figure 3c). This means that the number of new genes increases as new sequenced genomes are introduced into the pangenome, while the inclusion of new sequenced genomes does not substantially alter the core genome; an indication of the incremental genetic diversity that exists between the studied strains (Figure 3d).

**Figure 4:**
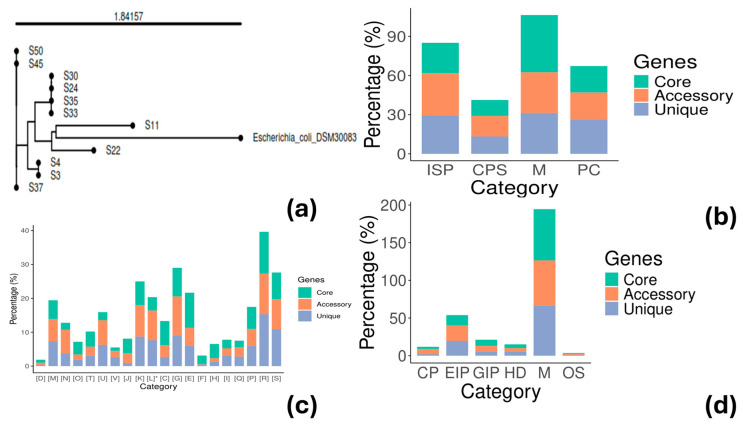
Genetic analysis of the *E. coli* strains: (**a**) SNP-based phylogenetic analysis of the studied *E. coli* strains, including the reference *E. coli* DSM30083 strain; (**b**) General COG-based functional categories of genes found in the core and accessory-unique genome; (**c**) COG-based functional annotation of genes found in the core and accessory-unique genome; and (**d**) KEGG-based functional categories of genes found in the core and accessory-unique genome. [D] Cell cycle control, cell division, chromosome partitioning; [M] cell wall/membrane/envelope biogenesis; [N] cell motility; [O] posttranslational modification, protein turnover, chaperones; [T] signal transduction mechanisms; [U] intracellular trafficking, secretion, and vesicular transport; [V] defense mechanisms; [J] translation, ribosomal structure, and biogenesis; [K] transcription; [L]* replication, recombination, and repair; [C] energy production and conversion; [G] carbohydrate transport and metabolism; [E] amino acid transport and metabolism; [F] nucleotide transport and metabolism; [H] coenzyme transport and metabolism; [I] lipid transport and metabolism; [Q] secondary metabolites biosynthesis, transport, and catabolism; [P] inorganic ion transport and metabolism; [R] general function prediction only; [S] function unknown. [L]*, the functional categories have been expanded, and the letter [X] has been used to denote phage-derived proteins, transposases, and other mobilome components. These proteins were initially included in the [L] category. ISP, Information Storage and Processing; CPS, Cellular Processes and Signaling; M, Metabolism; PC, Poorly Characterized; CP, Cellular Processes; EIP, Environmental Information Processing; GIP, Genetic Information Processing; Human Diseases; and OS, Organismal Systems.

Phylogenetic analysis of the *E. coli* genomes was conducted with the kSNP v3.0 tool for creating a single-nucleotide polymorphism (SNP)-based phylogenetic tree, which provides more discriminatory power. The analysis confirmed the existence of five different groups of strains: a. S3, S4 (2 strains); b. S11 (one strain); c. S22 (one strain); d. S24, S30, S33, S35 (4 strains); and e. S37, S45, S50 (3 strains), supporting the genetic diversity results previously observed (Figure 4a). Functional annotation with GOG showed different categories/subsystems between the core and accessory genome (Figure 4b). The genes found in the core genome were mainly related to metabolism classification (43.94%) (Figure 4b), such as energy production and conversion, metabolism of carbohydrates, amino acids, lipids, nucleotides, and coenzymes (Figure 4c), and less to information storage and processing (23.33%) or cellular processes and signaling (12.37%) (Figure 4b). The opposite was true for the accessory and unique genes where metabolism accounted only for 31.15% and 31.28%, respectively, while information storage and processing accounted for 32.53% and 29.20%, respectively (Figure 4b). The latter classification included cell wall/membrane biogenesis, cell motility, defense mechanisms, transcription, and phage-derived proteins, transposases, and other mobilome components (Figure 4c). KEGG functional annotation provided complementary information (Figure 4d). Core genes were mostly associated with metabolism (around 70% of the genes found in the core genome). Approximately 41% (accessory genome) and 34% (unique genome) of the genes were assigned to functions related to cellular processes, environmental information processing, genetic information processing, and human diseases.

### 3.4. Antimicrobial Resistance

**Figure 5:**
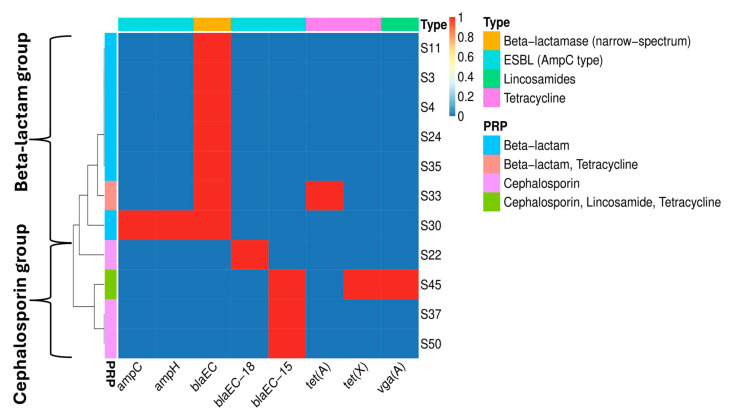
In-silico screening of the whole-genome-sequenced *E. coli* strains (draft genomes) for the identification of ARGs. Red color, presence; blue color, absence. The red color indicates the presence of the gene (genes in columns) in a genome (strains in rows) and, based on the color on the top of the graph, it shows to which antibiotic it confers resistance (type). The colors located on the left-hand side show the predicted resistance phenotype (PRP) of each strain as predicted by the bioinformatic analysis.

The *E. coli* strains were screened for the presence of ARGs related to different classes of antibiotics (Figure 5). The results revealed, in general, the existence of two groups. The first was the “beta-lactam” group, including the strains S3, S4, S11, S24, S30, S33, and S35, which harbored the gene *bla*_EC_. This gene provides resistance to the beta-lactam antibiotic class (narrow-spectrum). The second group was the “cephalosporin” group, in which variants of the *bla* gene were detected in the S22 (*bla*_EC-18_), S37 (*bla*_EC-15_), S45 (*bla*_EC-15_), and S50 (*bla*_EC-15_) strains. These genes confer resistance to the antibiotic class of cephalosporins. Two strains possessed genes related to tetracycline resistance [gene: *tet*(A), strain: S33; gene: *tet*(X), strain: S45]. Strain S45 also had a gene related to lincosamide resistance [gene: *vga*(A), strain: S45], which also confers resistance to other antimicrobials. AmpC type ESBL resistance (beta-lactams-cephalosporins) was detected in one strain (S30). Finally, all strains harbored several multidrug efflux pump systems (*acrA*, *acrB*, *acrD*, *acrE*, *acrF*, *emrA*, *emrB*, *emrD*, *emrK*, *emrY*, *mdfA*, *mdtABC*, *mdtEF*, *mdtM*, *mdtNOP*, and *tol*C) and regulators (*acrS*, *baeRS*, *cpxAR*, *emrR*, *evgAS*, *marAR*, *HNS*, and *crp*). The *emrD* is a multidrug transporter from the Major Facilitator Superfamily (MFS), which confers resistance to phenicol antibiotics, disinfecting agents, and antiseptics and may play a crucial role in biofilm development [67,68].

Based on the in-silico results related to the discovery of ARGs, two representative strains (S30 and S45) from each group were selected and checked phenotypically for their susceptibility to different classes of antibiotics using the mastitis plate of the Sensititre™ MIC platform (Table 2). The output of the AST supported the results previously found. The strain S30 presented (a) resistance to ceftiofur (cephalosporin), cephalothin (cephalosporin), oxacillin (narrow-spectrum beta-lactam), and sulphadimethoxine (sulfonamide); (b) intermediate resistance to ampicillin (penicillin), penicillin (penicillin), and pirlamycin (lincosamide); and (c) susceptibility to erythromycin (macrolide), penicillin/novobiocin (a combination of penicillin and aminocoumarin), and tetracycline (tetracycline). The strain carried genes (*bla*_EC_, *ampC*, *ampH*) and multidrug proteins (*acrF*, *mdtM*), which provide resistance to the antibiotic classes of beta-lactams/penicillins, cephalosporins, and lincosamides (*mdtM*).

Strain S45 was susceptible to most of the antibiotics and only exhibited resistance to sulphadimethoxine (sulfonamide) and tetracycline (tetracycline). The gene *tet*(X), related to tetracycline resistance, was found in the strain. Although the strain harbored genes that confer resistance to cephalosporins (*bla*_EC-15_) and lincosamides [*vga*(A)], no such resistance phenotype was observed. The two strains shared the same profile (resistant) to sulphadimethoxine (sulfonamide), although no genes (*sul1*, *sul2*, *sul3*, *sul4*) associated with this profile were detected in the genomes of both strains. This could be attributed to the function of some of the numerous efflux pumps found in their genome, which confer resistance to a broad spectrum of chemically unrelated substrates, including antibiotics, or to the inability of the AMR databases to detect these genes because of the presence of fragmented genes, resulting in a low detection score. Consequently, in-silico screening for ARGs should be accompanied by laboratory phenotypic tests for reliable determination of the AMR of a strain, especially when draft (short-reads) genomes are analyzed.

The strain S30 showed resistance to cephalosporins (beta-lactams) and sulfonamides, including intermediate resistance to a different class of antibiotics like lincosamides. This strain was an AmpC type ESBL which describes a specific type of AMR including the production of ESBLs and AmpC beta-lactamases, constituting a significant concern for public health. AmpC beta-lactamases and ESBLs both contribute to cephalosporin resistance, but they have different properties. ESBLs are inactivated by beta-lactamase inhibitors, commonly used in combination with beta-lactam antibiotics to overcome resistance, whereas AmpC enzymes remain unaffected.

### 3.5. Virulence Factors and Mobile Genetic Elements

The search for virulence factors of the eleven *E. coli* draft genomes revealed the following (Table 3):

i. All strains were negative for *stx* (shigatoxin—Shiga Toxin-producing *E. coli* (STEC) strains or EHEC), LAA PAI (Locus of Adhesion and Autoaggregation Pathogenicity Island), primarily found in LEE-negative (Locus of Enterocyte Effacement) STEC strains, *estA* (the gene encodes a heat-stable enterotoxin in ETEC), *eltAB* (the genes encode the A and B subunits of a heat-labile enterotoxin in ETEC), and pEAF/*bfp* [EPEC adherence factor plasmid, bundle-forming pilus operon, and plasmid-encoded regulator (*perABC*) gene cluster, which constitutes the adherence factor in typical EPEC].
ii. All strains were *eae*-positive (the adhesion factor of EPEC and EHEC). The intimin-encoding gene *eae* is crucial in the production of the attaching and effacing (A/E) lesions (LEE PAI). The *esp* genes, such as *espL*, *espR*, *espX*, and *espY*, which encode proteins that are secreted by the Type III secretion system and are involved in various steps of the infection process, including attaching to and damaging host cells, were also identified. These genes are crucial for the virulence of EPEC and EHEC.
iii. The LEE PAI was partially identified in all strains; it is found in EPEC and EHEC. The PAI plays an important role in the attachment and effacing (A/E) lesion formation on intestinal epithelial cells. Partially identified means that the PAI was not completely detected (only 15 to 39% of the PAI was identified). The examined genomes were not complete but in contigs/scaffolds, meaning that several genes could be fragmented and, therefore, difficult to be detected and recorded by the program, as the identification value was far below the software’s threshold.
iv. The strains S3, S11, S22, S37, S45, and S50 were positive to the presence of the ETT2 PAI and its regulator (*etrA*) found in EHEC and STEC, but also in atypical EPEC, EAEC, and ExPEC strains. The PAI encodes a type III secretion system (T3SS) known as ETT2 T3SS, which is involved in the production of several effectors and regulatory proteins but also extends its function by affecting the expression of other virulence genes outside the PAI [69,70,71].
v. The ETT2-negative strains (S4, S24, S30, S33, and S35) possessed alternative mechanisms of adherence and aggregation such as the *fim* gene cluster (*fimABCDEFGHI*), *papCD* genes, and *focC* gene (except for S4).
vi. All strains were *astA*-positive (the gene encodes a heat-stable enterotoxin in EHEC, EAEC, and atypical EPEC).
vii. All strains shared ExPEC-like genetic determinants such as *ybtP* (iron), *irp1* and *irp2* (invasins), *hlyE* (toxin), *fimF*, *fimG*, *fimH*, *yagVWXYZ/ecpABCDE*, *ykgK/ecpR*, *papCD* and *focC* (adhesins), and *ompA*, *ompC*, *ompD*, *ompF*, *ompG*, and *ompT* (serum resistance proteins).
viii. The strains S24, S30, S33, and S35 were *aatA*-positive (the gene encodes a dispersing protein).
ix. The strain S3 was PAI IV- and HPI-positive. The first PAI contains various VFs related to inflammation, adhesion, colonization, and protein secretion (type I secretion system—T1SS). This PAI is characterized by its proximity to tRNA-encoding genes and the presence of integrase which facilitates its movement within the prokaryotic genome and/or between other microbes through horizontal gene transfer [72]. The second PAI is involved in iron uptake through the production of a siderophore (yersiniabactin), enhanced autophagy, and other virulence mechanisms (flagellum-mediated motility) [73].
x. The invasin *ibeB*, detected in all strains, plays a crucial role in bacterial invasion. It is frequently associated with other VFs such as *ibeA* (the gene was not detected) and *ompA*, causing tissue penetration, including of host cells coating the blood–brain barrier, indicating the high pathogenicity potential of the strains [10].

**Table 3:** VFs and MGEs found in the whole-genome-sequenced *E. coli* strains (draft genomes).

Strain ID	Virulence Islands ^1^	Iron	Protease	Adhesins	Invasins	Toxins
S3	PAI IV (68.42%), HPI (66.67%), EET2 (70.27%) plus *etrA*, and LEE (21.95%) plus *eae*	*fecA-E* ^2^, *ybtP*	*ompACDFGT*	f*imA-I*, *fdeC*, *yagV-Z*/*ecpA-E*, *ykgK*/*ecpR*, *nlpADEI*, *yehA-D*, *lpfA*, *papCD*, and *espLRXY*	*csgA-G*, *aslA*, *fyuA*, *gadBCEWX*, *ibeB*, *irp1*, *irp2*, and *hha*	*hlyE* and *astA*
S4	LEE (21.95%) plus *eae*	*fecA-E*, *ybtP*	*ompACDFGT*	f*imA-I*, *fdeC*, *yagV-Z*/*ecpA-E*, *ykgK*/*ecpR*, *nlpADEI*, *yehA-D*, *lpfA*, *papCD*, and *espLRXY*	*csgA-G*, *aslA*, *fyuA*, *gadBCEWX*, *ibeB*, *irp1*, *irp2*, and *hha*	*hlyE* and *astA*
S11	EET2 (97.30%) plus *etrA* and LEE (39.02%) plus *eae*	**-**	*ompACDFG*	f*imA-I*, *fdeC*, *yagV-Z*/*ecpA-E*, *ykgK*/*ecpR*, *nlpADEI*, *yehA-D*, *lpfA*, *papCD*, *focC*, and *espLRXY*	*csgA-G*, *aslA*, *chuU-W*, *gadBCEWX*, *ibeB*, *hha*, and *tra*	*hlyE* and *astA*
S22	EET2 (70.27%) plus *etrA* and LEE (14.63%) plus *eae*	-	*ompACDFG*	*aaiADF*, f*imA-I*, *fdeC*, *faeC-G*, *yagV-Z*/*ecpA-E*, *ykgK*/*ecpR*, *nlpADEI*, *yehA-D*, *papCD*, *focC*, *upaG*/*ehaG*, and *espLRXY*	*csgA-G*, *gadBCEWX*, *ibeB*, *hha*, and *tra*	*hlyE* and *astA*
S24	LEE (17.07%) plus *eae*	*fecA-E*	*ompACDFGT*	*aatA*, f*imA-I*, *nlpADEI*, *yehA-D*, *lpfA*, *papCD*, *focC*, and *espLRXY*	*csgA-G*, *gadBCEWX*, *ibeB*, *hha*, and *tra*	*hlyE* and *astA*
S30	LEE (17.07%) plus *eae*	*fecA-E*	*ompACDFGT*	*aatA*, f*imA-I*, *nlpADEI*, *yehA-D*, *lpfA*, *papCD*, *focC*, and *espLRXY*	*csgA-G*, *gadBCEWX*, *ibeB*, *hha*, and *tra*	*hlyE* and *astA*
S33	LEE (17.07%) plus *eae*	*fecA-E*	*ompACDFGT*	*aatA*, f*imA-I*, *nlpADEI*, *yehA-D*, *lpfA*, *papCD*, *focC*, and *espLRXY*	*csgA-G*, *gadBCEWX*, *ibeB*, *hha*, and *tra*	*hlyE* and *astA*
S35	LEE (17.07%) plus *eae*	*fecA-E*	*ompACDFGT*	*aatA*, f*imA-I*, *nlpADEI*, *yehA-D*, *lpfA*, *papCD*, *focC*, and *espLRXY*	*csgA-G*, *gadBCEWX*, *ibeB*, *hha*, and *tra*	*hlyE* and *astA*
S37	EET2 (70.27%) plus *etrA* and LEE (17.07%) plus *eae*	*fecA-E*	*ompACDFG*	f*imA-I*, *fdeC*, *yagV-Z*/*ecpA-E*, *ykgK*/*ecpR*, *nlpADEI*, *yehA-D*, *lpfA*, *papCD*, *upaG*/*ehaG*, and *espLRXY*	*csgA-G*, *aslA*, *gadBCEWX*, *ibeB*, *hha*, and *tra*	*hlyE* and *astA*
S45	EET2 (70.27%) plus *etrA* and LEE (17.07%) plus *eae*	*fecA-E*	*ompACDFG*	f*imA-I*, *fdeC*, *yagV-Z*/*ecpA-E*, *ykgK*/*ecpR*, *nlpADEI*, *yehA-D*, *lpfA*, *papCD*, *upaG*/*ehaG*, and *espLRXY*	*csgA-G*, *aslA*, *gadBCEWX*, *ibeB*, and *hha*	*hlyE* and *astA*
S50	EET2 (70.27%) plus *etrA* and LEE (17.07%) plus *eae*	*fecA-E*	*ompACDFG*	f*imA-I*, *fdeC*, *yagV-Z*/*ecpA-E*, *ykgK*/*ecpR*, *nlpADEI*, *yehA-D*, *lpfA*, *papCD*, *upaG*/*ehaG*, and *espLRXY*	*csgA-G*, *aslA*, *gadBCEWX*, *ibeB*, and *hha*	*hlyE* and *astA*

^1^ The number inside the parenthesis refers to the percentage of the PAI identified. ^2^ Intermediate letters are also included.

The results showed that *E. coli* strains carry genes similar to those of ExPEC, which cause infections outside the intestinal tract, such as iron scavenging, host attachment and colonization, immune evasion, and production of toxins and serum resistance proteins [3,10,74]. In addition, the ETT2 PAI, found in all strains, has a significant role in their motility, biofilm formation, adhesion, invasion, serum resistance, survival, and interference with the host’s immune system. This PAI is particularly important for the ExPEC’s virulence, even when ETT2 is not intact [69,70,71]. PAIs are deemed parts of the group of mobile genetic elements, suggesting that they can move within a genome and/or between bacteria [72]. All strains were positive for *hlyE* (hemolysin E) or the *fec* (ferric dicitrate iron acquisition) system (another ExPEC-like characteristic, which enhances their virulence), which is a pore-forming toxin disrupting red blood cells and other cells, but also can promote colonization, motility, and biofilm formation [3,75].

The isolated strains presented genetic properties similar to atypical EPEC (aEPEC). aEPEC are distinguished by their possession of the *eae* gene for A/E lesion formation and the lack of the *bfp* gene for bundle-forming pili, placing them in a separate genetic group compared to typical EPEC (tEPEC). This difference affects their adherence patterns and virulent characteristics. More specifically, the strains showed the following properties reinforcing their similarity to aEPEC [76,77]:

- **pEAF/*bfp*—negative:** The EPEC adherence factor (EAF) plasmid and *bfpA* gene are both absent in aEPEC, which encode the bundle-forming pili, a protein involved in localized adherence to host cells.
- ***eae*—positive:** The aEPEC, similar to tEPEC, harbors the *eae* gene, which is a key gene for the formation of A/E lesions, a crucial virulence factor of EPEC.
- ***stx*—negative:** The presence of *stx* gene is the trademark of STEC. Although some EPEC may possess the shiga toxin gene, the aEPEC do not produce shiga toxins.
- **Genetic similarity to STEC:** The aEPEC are genetically closer to STEC than tEPEC, showing similarities in serotypes and other epidemiological aspects. The five serotypes identified in this work (O179:H40, O169:H46, O18ac:H7, ONT:H26, and O107:H27), all were STEC serotypes.
- **Diversity in genetic background:** The aEPEC displays genetic diversity with some strains exhibiting closer relationships to other *E. coli* pathotypes such as ETEC or ExPEC (Figure 1b).

While aEPEC lacks the pEAF/*bfp* many strains may carry the ETT2 PAI. Apart from S4, all other strains possessed this pathogenicity island. The ETT2 PAI is a specific genomic region containing genes that contribute to virulence and pathogenicity. Consequently, the presence of this PAI in the aEPEC implies its pathogenic potential. ETT2-negative strains such as the S4 can harbor alternative mechanisms of adherence and aggregation. These VFs are often encoded on plasmids or other PAIs. For instance, the LEE PAI plus the *eae* gene or *pap* encoding P fimbriae, *sfa* encoding S fimbriae, *foc* encoding F1C fimbriae, and *fim* gene cluster encoding type 1 pili help aEPEC strains colonize and cause disease [78].

The analysis showed the presence of a high number of Col and Inc plasmid groups (Table 4). The strains S24, S30, S33, and S35 were characterized by the presence of seven to nine plasmids compared to the rest, in which only zero to two plasmids were detected. This difference probably contributes to the genetic diversity of this group (Figure 4a). Plasmids frequently carry virulence or AMR genes. From the Inc group, the majority of the plasmids were of the IncF family. This kind of plasmid is frequently conjugative, demonstrating its capacity to distribute resistance. Notably, the IncF family were mainly found in the ST4977 group (S24, S30, S33, and S35), which included the S30 strain resistant to different antibiotics. Similar results have been observed in other studies as well [3,79]. In addition, *E. coli* strains possessing plasmids like IncF or IncI1 have been reported as high-risk clones [80,81]. Col plasmids are frequently found in *E. coli* and are associated with the production of proteins like bacteriocins that inhibit the bacterial growth of other microorganisms (e.g., colicin E) [82]. The presence of a plethora of MGEs signifies the plasticity of the *E. coli* genome and the high potential of acquiring other VFs and ARGs through horizontal gene transfer. For example, the *aaiA* gene found in some *E. coli* strains is a virulence factor, specifically encoding a component of the type VI secretion system (T6SS), which is associated with the aggregative adherence phenotype, a trademark of EAEC. AggR is a transcriptional regulator that plays a crucial role in virulence by activating the expression of numerous genes involved in adherence, biofilm formation, and potential toxin production. It is a key factor that defines typical EAEC strains. In addition, the genes *aaiA* (along with *aaiC* and *aaiG*) and *aatA* (the gene encoding a protein involved in aggregation) are important for identifying typical and atypical EAEC. The AggR and *aatA* are frequently found on plasmids [83,84]. Furthermore, the *aatA* and *astA* genes, both found in the isolated *E. coli* strains, are virulent factors that contribute to the bacterium’s ability to cause diarrheal disease and may also facilitate bacterial colonization of the intestine. The *astA* gene encodes a heat-stable enterotoxin that is produced by some *E. coli* strains, including EHEC, EAEC, and aEPEC [85,86].

## 4. Conclusions

The outputs of this work showed that *E. coli* strains isolated from raw sheep milk were identified as hybrid *E. coli*, possessing many MGEs, a fact that highlights the genomic plasticity of this species. The strains that were identified in the present work were genetically closer to aEPEC, sharing ExPEC-like genetic characteristics as well. Hybrid *E. coli* strains have been identified in several studies [3,10,12,87]. Moreover, different serotypes circulating within Greek dairy sheep were identified, which are reported for the first time (e.g., ST4977). aEPEC strains are more closely related to STEC, and like STEC, these strains appear to be emerging pathogens, because they possess VFs that can cause disease. aEPEC can cause illness in humans, and, therefore, they can be considered zoonotic foodborne pathogens. Hybrid *E. coli* strains, detected in raw sheep milk and isolated in the current study, carry various VFs and ARGs (genetic variability), making their categorization and treatment a difficult task; consequently, they threaten public health. The strains selected for AST harbored ARGs to compensate for the toxic effect of different antibiotic classes. ARGs, like virulence genes, are frequently associated with MGEs (PAIs, plasmids, etc.), which is an additional public health concern as these genetic traits are easily transferred to other microbes.

Typically, 60–70% of the examined raw sheep milk samples are found positive for the presence of *E. coli*, usually at low to moderate concentration levels (100–1000 cfu/mL). In a work carried out in Central Italy, the authors tested 372 bulk tank sheep milk samples from 87 farms and found that approximately 61% were positive to *E. coli*. Around 75% of the positive samples had a contamination level below 100 cfu/mL [88]. Better hygiene practices during milking (e.g., fast cooling, clean udders, and avoidance of hand contact) correlated with lower contamination levels [88]. In another study, it was reported that around 67% of the raw sheep milk samples were positive to *E. coli*, with contamination levels mostly between 100 and 1000 cfu/mL, only some exceeding 1000 cfu/mL [89]. In addition, pathogenic strains such as STEC/O157:H7 have been found in raw sheep milk. A meta-analysis study reported a STEC prevalence in raw sheep milk and cheeses made from sheep milk ca. 4.8% and 2.8%, on average, respectively [90]. In a Spanish study with raw milk from ewes, three samples were positive to *E. coli* O157:H7, and the isolates harbored virulence genes such as *stx1*/*stx2*, *eaeA*, and *ehxA* [91]. Therefore, although the overall counts of *E. coli* in raw sheep milk are usually low, the presence of pathogenic strains, e.g., STEC including *E. coli* O157:H7, even at low levels, poses a public health risk. Routine testing for generic *E. coli* is needed because it serves as a hygiene indicator. The examination of raw sheep milk specifically for pathogenic *E. coli* serotypes is warranted, considering its intended use, i.e., milk is mainly directed to the manufacturing of dairy products such as artisanal cheeses.

This work highlights the utility of WGS as a tool to better describe and assess the zoonotic potential of *E. coli* strains cultured from raw sheep milk samples. WGS showed varied *E. coli* residents consisting of different serotypes, STs, cgSTs, VFs, and AMR profiles. The data revealed that raw sheep milk could be a potential reservoir of zoonotic *E. coli* strains harboring easily exchangeable virulence and resistance genes.

## Figures and Tables

**Table 1:** Genotyping of the eleven whole-genome-sequenced *E. coli* strains (draft genomes).

Strain ID ^1^	Phylogroup	O:H Serotype	MLST (Achtman)	cgMLST	FimType	CHType	Human Pathogen
S3	A	O179:H40	ST10	23273	fimH137	fumC11	Yes (0.940) ^2^
S4	A	O179:H40	ST10	23273	fimH137	fumC11	Yes (0.941)
S11	E	O169:H46	ST1131	34239	fimH31	fumC23	Yes (0.938)
S22	B1	O18ac:H7	ST351	148610	fimH31	fumC95	Yes (0.942)
S24	A	ONT:H26	ST4977	56618	fimH27	fumC11	Yes (0.847)
S30	A	ONT:H26	ST4977	56618	fimH27	fumC11	Yes (0.933)
S33	A	ONT:H26	ST4977	56618	fimH27	fumC11	Yes (0.932)
S35	A	ONT:H26	ST4977	56618	fimH27	fumC11	Yes (0.842)
S37	A	O107:H27	ST10	23653	fimH54	fumC11	Yes (0.934)
S45	A	O107:H27	ST10	23653	fimH54	fumC11	Yes (0.932)
S50	A	O107:H27	ST10	23653	fimH54	fumC11	Yes (0.874)

^1^ Strains S16, S19, S25, and S40 were excluded because the quality metrics of their genome assemblies were below the respective threshold. ^2^ Whether the microorganism is predicted as human pathogenic (yes or no) and the probability of being a human pathogen (inside the parenthesis).

**Table 2:** AST of the *E. coli* strains S30 and S45 using the Sensititre™ MIC platform with the Sensititre™ Mastitis CMV1AMAF Vet AST Plate.

Antibiotic/Drug	Abbreviation	S30	S45	Antibiotic Class
Ampicillin	AMP	I	S	Penicillin (Beta-lactam)
Ceftiofur	XNL	R	S	Cephalosporin (Beta-lactam)
Cephalothin	CEP	R	S	Cephalosporin (Beta-lactam)
Erythromycin	ERY	S	S	Macrolides
Oxacillin + 2% NaCl	OXA+	R	S	Penicillin (Beta-lactam)
Penicillin	PEN	I	S	Penicillin (Beta-lactam)
Penicillin/Novobiocin	P/N	S	S	Beta-lactam/Aminocoumarin
Pirlamycin	PIRL	I	S	Lincosamide
Sulphadimethoxine	SDM	R	R	Sulfonamide
Tetracycline	TET	S	R	Tetracyclines

Symbols: R, resistant; I, intermediate resistance; S, susceptible.

**Table 4:** Plasmids and phages found in the whole-genome sequenced *E. coli* strains (draft genomes).

Strain ID	Plasmids	Phages
S3	Col156	-
S4	-	-
S11	ColpVC and IncFII(pCoo)	Lambdavirus and peduovirus
S22	IncFII(pCoo), IncFIA, and IncFIB(AP001918)	Lambdavirus and peduovirus
S24	ColpVC, Col(MG828), Col156, Col8282, IncFII(29)_pUTI89, IncI1(Alpha), and Col(KPHS6)	-
S30	ColpVC, Col(MG828), Col156, Col8282, IncFII(29)_pUTI89, IncI1(Alpha), and Col(KPHS6)	-
S33	ColpVC, Col(MG828), Col156, Col8282, IncFII(29)_pUTI89, IncI1(Alpha), Col(KPHS6), Col440I, and ColRNAI	Lambdavirus
S35	ColpVC, Col(MG828), Col156, Col8282, IncFII(29)_pUTI89, IncI1(Alpha), Col(KPHS6), and rep33_rep(pSMA198)	-
S37	IncY	-
S45	rep19b_repA(SAP105A) and rep5b_rep(pUR2355)	-
S50	ColpVC	-

## Data Availability

The whole-genome sequencing data have been deposited at GenBank (NCBI) under accession (BioProject) number PRJNA1294326 (https://www.ncbi.nlm.nih.gov/, accessed on 24 May 2025).

## Supplementary Materials

The following supporting information can be downloaded at: https://www.mdpi.com/article/10.3390/vetsci12080744/s1, Table S1. Quality metrics of the genome assembly of fifteen whole-genome-sequenced *E. coli* strains (draft genomes) and one reference strain; Table S2. Genome characteristics and annotations of the eleven whole-genome-sequenced *E. coli* strains (draft genomes) and one reference strain.

## Abbreviations

The following abbreviations are used in this manuscript:

VFs	Virulence Factors
AMR	Antimicrobial Resistance
CNS	Coagulase-Negative Staphylococci
SCM	Subclinical Mastitis
CM	Clinical Mastitis
STs	Sequence Types
MEcS	Mastitis *Escherichia coli* Strains
MGEs	Mobile Genetic Elements
IPEC	Intestinal Pathogenic *Escherichia coli*
ExPEC	Extraintestinal Pathogenic *Escherichia coli*
EAEC	Enteroaggregative *Escherichia coli*
EPEC	Enteropathogenic *Escherichia coli*
EIEC	Enteroinvasive *Escherichia coli*
EHEC	Enterohemorrhagic *Escherichia coli*
ETEC	Enterotoxigenic *Escherichia coli*
STEC	Shiga Toxin-producing *Escherichia coli* strains
BHI	Brain Heart Infusion
SCC	Somatic Cell Count
EUCAST	European Committee on Antimicrobial Susceptibility Testing
CLSI	Clinical and Laboratory Standards Institute
AST	Antimicrobial Susceptibility Testing
ANI	Average Nucleotide Identity
MLST	Multi-Locus Sequence Typing
cgMLST	core-genome Multi-Locus Sequence Typing
CGE	Center for Genomic Epidemiology
iTOL	Interactive Tree of Life
ARGANNOT	Antibiotic Resistance Gene Annotation
CARD	Comprehensive Antibiotic Resistance Database
EcOH	*Escherichia coli* O-groups and H-types
NCBI	National Center for Biotechnology Information
TYGS	Type (Strain) Genome Server
GTDB-Tk	Genome Taxonomy Database Toolkit
MEGARes	Microbial Ecology Group Antimicrobial Resistances
IPGA	Integrated Prokaryotes Genome and pan-genome Analysis service
ARGs	Antimicrobial Resistance Genes
WGS	Whole-Genome Sequencing
CDS	Coding DNA Sequence
rRNA	Ribosomal RNA
tRNA	Transfer RNA
tmRNA	Transfer-Messenger RNA
dDDH	digital DNA–DNA Hybridization
BRIG	BLAST Ring Image Generator
BLAST	Basic Local Alignment Search Tool
COGs	Clusters of Orthologous Groups/Genes
SNPs	Single Nucleotide Polymorphisms
KEGG	Kyoto Encyclopedia of Genes and Genomes
ESBL	Extended-Spectrum Beta-Lactamase
MFS	Major Facilitator Superfamily
LEE	Locus of Enterocyte Effacement
LAA	Locus of Adhesion and Autoaggregation
PAI	Pathogenicity Island
