# Supplementary material for: Escherichia coli Strains Originating from Raw Sheep Milk, with Special Reference to Their Genomic Characterization, Such as Virulence Factors (VFs) and Antimicrobial Resistance (AMR) Genes, Using Whole-Genome Sequencing (WGS)
Source: Vet Sci. 2025 Aug 8;12(8):744. doi: 10.3390/vetsci12080744 (PMC12390150; doi:10.3390/vetsci12080744)
Supplement: Supplementary file 1 [file vetsci-12-00744-s001.zip › vetsci-3691811-supplementary.pdf]

Table S1. Quality metrics of the genome assembly of fifteen whole-genome sequenced *E. coli* strains (draft genomes) and one reference strain.

Strain ID (isolation time, dd.mm.yyyy)	Completeness (%)	Contamination (%)	Heterogeneity (%)	No. of contigs/scaffolds (≥300 bp)	N50 (bp) [1]	Genome size (bp)
S3 (08.05.2022)	99.97	0.10	0	96	153,848	4,725,589
S4 (08.05.2022)	99.97	0.10	0	2	4,517,476	4,717,828
S11 (06.06.2022)	99.97	0.33	0	136	221,657	5,003,699
**S16 (06.06.2022)**	**87.40**	1.50	**30**	**877**	**15,477**	4,655,651
**S19 (27.06.2022)**	99.93	**8.51**	0	**592**	170,680	**5,818,749**
S22 (27.06.2022)	99.93	0.10	0	116	166,190	4,876,137
S24 (04.07.2022)	99.48	0.06	0	114	98,591	4,804,944
**S25 (04.07.2022)**	**88.38**	0.66	**30**	**395**	37,409	**4,463,651**
S30 (01.08.2022)	99.30	0.11	0	108	100,546	4,775,225
S33 (01.08.2022)	99.67	0.12	0	151	146,320	4,911,813
S35 (05.09.2022)	99.53	0.08	0	106	98,854	4,835,616
S37 (05.09.2022)	99.97	0.04	0	123	147,602	4,770,204
**S40 (05.09.2022)**	98.58	1.28	0	**764**	**15,687**	5,293,133
S45 (19.09.2022)	99.58	0.51	0	156	99,928	4,991,789
S50 (31.10.2022)	99.05	0.04	0	147	75,368	4,529,899
Ref. st. [2]	99.97	0.39	0	2	4,903,501	4,903,501

[1] The sum of the lengths of all contigs of size N50 or longer contain at least 50% of the total genome sequence.

[2] Reference strain: *Escherichia coli* DSM30083.

Data in bolds do not meet the quality threshold of the respective metric.

Table S2. Genome characteristics and annotations of the eleven whole-genome sequenced *E. coli* strains (draft genomes) and one reference strain.

Strain ID [1]	No. of CDSs [3]	No. of genes	GC content (%)	Repeat region	rRNA [3]	tRNA [3]	tmRNA [3]
S3	4,374	4,461	50.62	2	6	80	1
S4	4,361	4,452	50.55	2	5	85	1
S11	4,596	4,686	50.72	2	4	85	1
S22	4,553	4,644	50.59	4	4	86	1
S24	4,487	4,566	50.67	3	3	76	- [4]
S30	4,455	4,535	50.72	3	3	76	1
S33	4,578	4,665	50.77	3	9	77	1
S35	4,511	4,585	50.51	3	1	73	- [4]
S37	4,463	4,553	50.55	1	5	84	1
S45	4,680	4,758	49.33	2	3	74	1
S50	4,212	4,288	50.60	1	2	74	- [4]
Ref. st. [2]	4,708	4,819	50.64	2	22	88	1

[1] Strains S16, S19, S25, and S40 have been excluded because the quality metrics of their genome assembly was below the respective threshold.

[2] Reference strain: *Escherichia coli* DSM30083.

[3] CDSs, Coding DNA Sequences; rRNA, ribosomal RNA; tRNA, transfer RNA; tmRNA, transfer-messenger RNA.

[4] Not reported/found by the PROKKA annotation tool.
